# Extensive disseminated cysticercosis: a case report in Yunnan province, China

**DOI:** 10.1186/s12879-019-4172-3

**Published:** 2019-06-17

**Authors:** Xin-Zhong Zang, Huan-Zhang Li, Men-Bao Qian, Ying-Dan Chen, Chang-Hai Zhou, Hong-Kun Liu, Yu-Hua Liu, Shi-Zhu Li

**Affiliations:** 1 National Institute of Parasitic Diseases, Chinese Center for Disease Control and Prevention, Key Laboratory of Parasite and Vector Biology, Ministry of Health, National Center for International Research on Tropical Diseases, Ministry of Science and Technology, WHO Collaborating Center for Tropical Diseases, Shanghai, 200025 China; 2 Dandong City Center for Disease Control and Prevention, Dandong, 118000 Liaoning province China; 3 Dali Prefectural Institute of Research and Control on Schistosomiasis, Dali, 671000 Yunnan province China

**Keywords:** Extensive disseminated cysticercosis, *Taenia solium*, Control

## Abstract

**Background:**

Cysticercosis is spreading all over the world and it is a major health problem in most countries of Latin America, Africa, and Asia. Extensive disseminated cysticercosis is relatively rare and fewer than 120 case have been reported in the worldwide. We reported a rare case of extensive disseminated cysticercosis in Yunan province, China.

**Case presentation:**

A rare case of extensive disseminated cysticercosis, in a 61-year-old male Chinese was detected from Yunnan province in 2018. Clinical and etiological examination was performed, as well as the epidemiological investigation.

**Conclusion:**

The life cycle of *T. solium* in the area where the case came from is complete. We expect this case could raise the attentions to the control of *Taenia solium* infection and subsequent cysticercosis there.

## Background

Cysticercosis refers to a parasitic infection caused by the larvae of the pork tapeworm *Taenia solium* [1, 2]*.* In 2010 and 2014, it was listed as one of the neglected tropical diseases (NTDs) and negligible zoonotic diseases (NZDs) by the World Health Organization (WHO) and the Food and Agriculture Organization of the United Nations (FAO), respectively [3]. Cysticercosis is spreading all over the world and it is a major health problem in most countries of Latin America, Africa, and Asia [1]. With the globalization and increasing exchanges between countries, more and more cases of cysticercosis have been reported in non-endemic areas, and cysticercosis was classified as an emerging infectious disease in some developed countries [1, 4]. In China, this disease is mainly endemic in southwestern areas, especially in Yunnan province where a fair portion of the residents have the habit of consuming raw or undercooked pork [4]. Cysticercosis can involve any tissue of the body, such as central nervous system, subcutaneous tissue, eyes and muscles [1, 2]. The central nervous system is most commonly afflicted in clinical cases [5, 6]. However, extensive disseminated cysticercosis is relatively rare and fewer than 120 case have been reported in the worldwide, the majority of which were in India [7–12]. Here, we reported a rare case of extensive disseminated cysticercosis from Yunan province, China. It is expected the control of *Taenia solium* infection and subsequent cysticercosis could be paid attentions there.

## Case presentation

In March 2018, a case with extensive disseminated cysticercosis was admitted in the medical department affiliated to the Institute of Research and Control on Schistosomiasis in Dali city. A 61-year-old male reported that his left lower extremity was treated because of fall fracture in a local hospital three years ago. During the examination, the doctor found multiple cysticercroid nodules in the intramuscular. Then, after the fracture was cured, the patient went to other hospitals and received three rounds of treatments for cysticercosis. However, we were unable to obtain the details of that treatment history of the patient. Recently, the patient felt mild headaches and dizziness. Then, he came to this hospital. A comprehensive investigation was carried out for the patient including symptom, physical examinations, imaging examinations, etiological examinations and epidemiological investigations.

Symptom and physical examinations: symptom and physical examinations were performed immediately after the patient was hospitalized. In addition to mild headaches and dizziness, the patient reported no other symptoms. No abnormality was found in physical examinations: 36.5°C of body temperature, 90 per min of pulse rate, 19 per min of breath rate, and 97/68 mmHg of blood pressure.

**Fig. 1:**
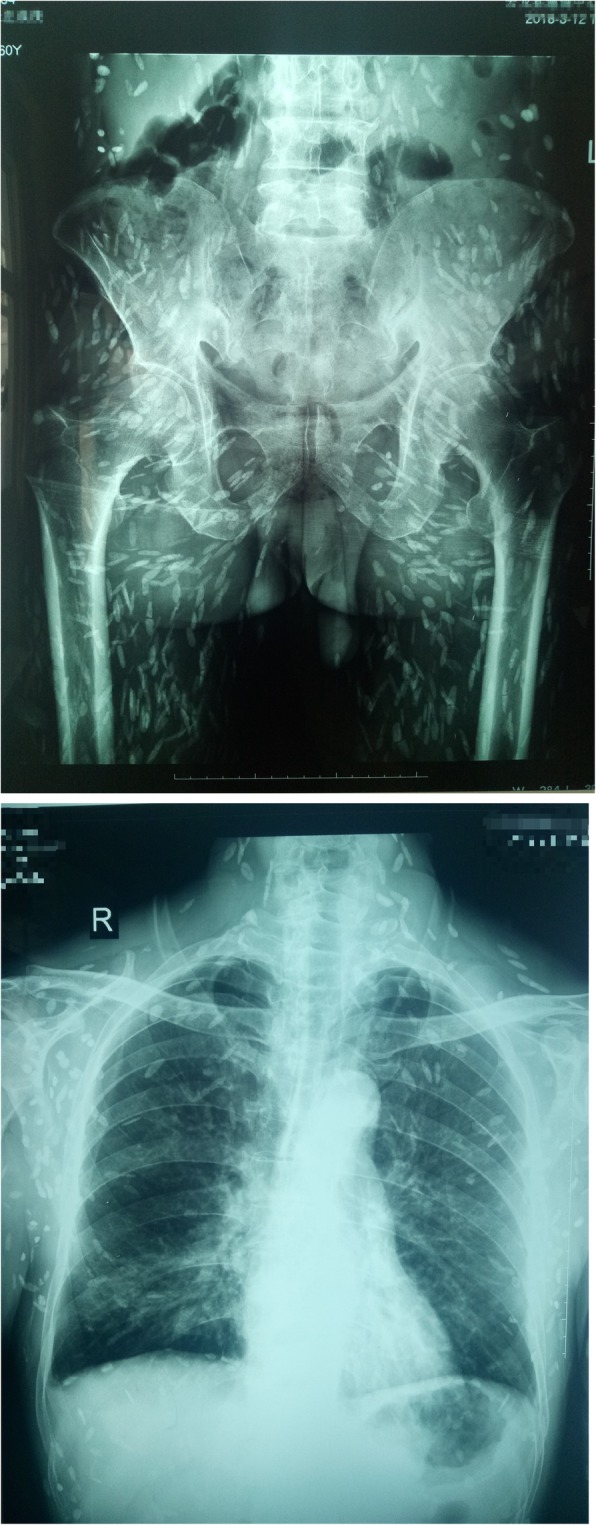
X-ray slices (Calcified foci were disseminated around the whole body, showing a “meteoroid”. The calcification of cysticerci presented as long spindles, long ovals, or elongated strips. Size of the calcareous cysticerci varied with clear outlines and some overlapped)

**Fig. 2:**
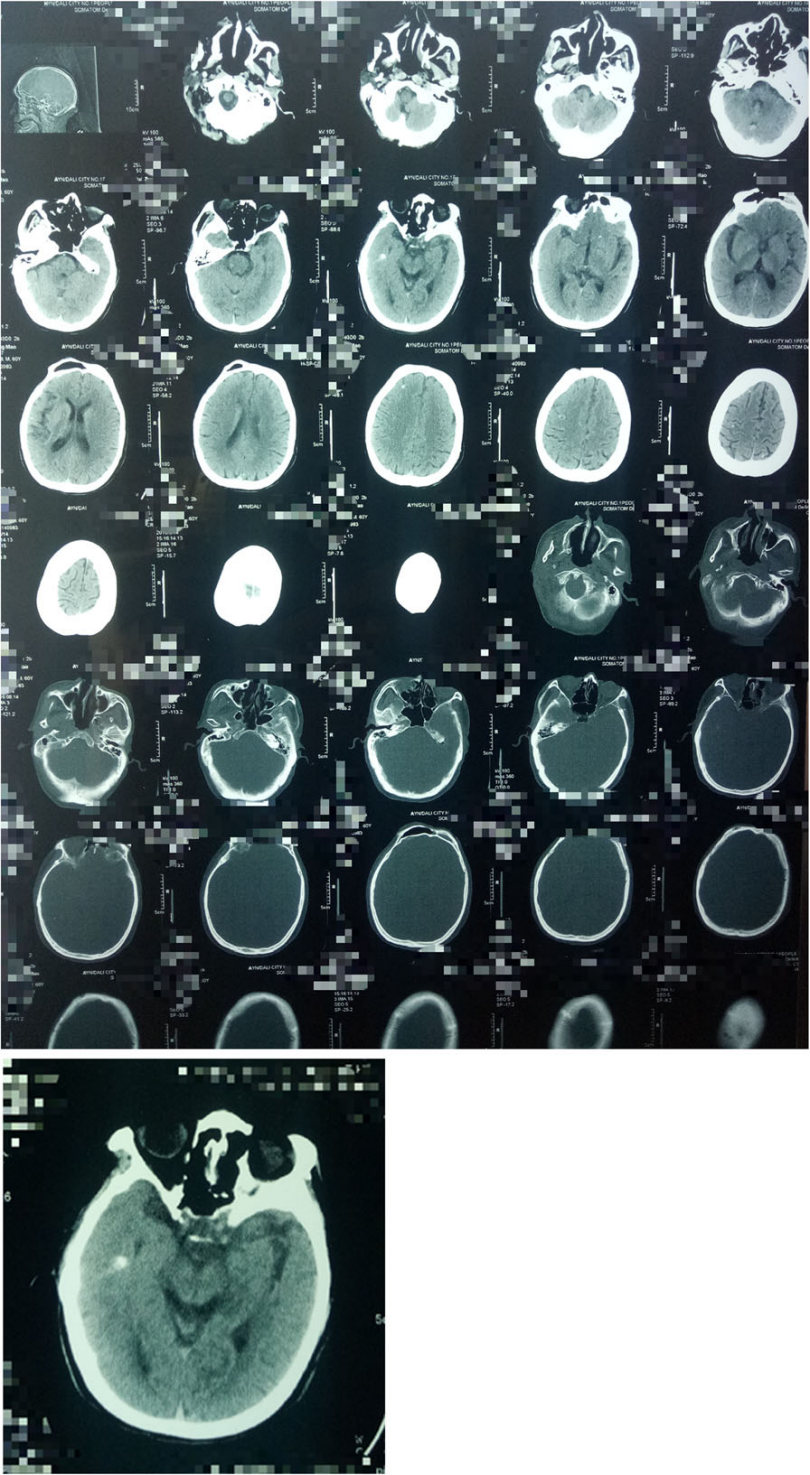
Brain CT scan (Bilateral nodules with multiple nodular calcifications were shown in bilateral cerebral hemispheres, with a diameter of approximately 1–2 mm)

Imaging examination: X-ray examination was done on March 12, 2018. Hundreds of calcification spots due to cysticercosis following the muscle planes were found (Fig. 1). Calcified foci were disseminated around the whole body, showing a “meteoroid”. The calcification of cysticerci presented as long spindles, long ovals, or elongated strips. Size of the calcareous cysticerci varied with clear outlines and some overlapped. The major axis of the calcification was mostly consistent with the direction of muscle fibers. Through the x-ray film, the patient had high systemic calcification density, with uneven distribution, namely high in limbs and less in trunk. On March 14, 2018, computed tomography (CT) scan was performed for the brain (Fig. 2). Bilateral nodules with multiple nodular calcifications were shown in bilateral cerebral hemispheres, with a diameter of approximately 1–2 mm, which met the diagnostic criteria for definite neurocysticercosis [13–15]. There existed no obvious abnormality in the binocular ultrasound. Finally, this case was diagnosed as mixed infection of neurocysticercosis and muscle cysticercosis.

Etiological examination: the patient’s blood and feces were examined. Antibody test against cysticercosis was performed with the kit (IgG) purchased from Shenzhen Kangbide Biotechnology Co., Ltd. Positive finding was presented. Fecal examination was done, but no eggs or proglottids of *T. solium* were found in the feces.

Epidemiological investigation: The patient grew up and mostly lived in Yunlong county, Dali city, where taeniasis and cysticercosis are endemic [4]. Pigs were raised by his family, and mostly self-slaughtered without any inspection and quarantine. Occasionally, pork was also sold in the community and surrounding markets. The patient reported that he had consumed pork containing cysticerci, but denied that he had a history of expelling worms or proglottids. His family members also denied cysticercosis-related symptoms and the history of expelling worms or proglottids. The sanitation was poor in patient’s community and raising pigs were common there. The patient told us a few people in the community had cysticercosis and taeniasis. Traditional toilets were available in the villages, but faeces were usually used as a farmyard fertilizer without harmless treatment. The drinking water in the community was mostly spring water or snowmelt. The patient and his neighbors liked to eating raw vegetables, and drinking unboiled water, and sometimes ingested undercooked pork. Based on the epidemiological information obtained, it is suspected that the life cycle of *T. solium* was complete in the community where the case lived. Then, we investigated the records of cysticercosis patients treated in the hospital from 2014 to 2018. It was found that there were another 13 cases of cysticercosis in the town where the patient was located. Thus, it is believed that  the life cycle of *T. solium* was complete in the community where the case lived.

Treatment and follow-up: Patients accepted cysticidal treatment based on the hospital’s guideline. Firstly, albendazole was administrated at 20 mg/kg daily for 10 days and then followed by praziquantel at 20 mg/kg daily for 6 days. Two months later, the patient followed the doctor’s advice for a second course of treatment as above. Because the patient did not feel any abnormal, the patient refused to a further imaging check due to economic factor.

## Discussion

Cysticercosis is a prevalent zoonosis in China. A human case of cysticercosis was first reported in China by Barnes in 1922 [16]. China has conducted two national surveys on parasitic diseases. About 1.3 million persons were estimated with *Taenia spp.* infection in 1988–1992, and then 0.55 million was estimated in 2001–2004 [4]. Although the prevalence of taeniasis in China is decreasing, taeniasis and cysticercosis are still endemic in the southwestern part of China. Such factors cause the endemicity of taeniasis and cysticercosis there as ingestion of undercooked pork and raw vegetables, drinking of unboiled water, using of traditional toilets, free-roaming of pigs, etc. [5, 17–19]. Based on our epidemiological survey, it is believed the life cycle of *T. solium* was complete in the community where the case came from. Therefore, if no effective measures are implemented, the endemicity will continue there.

Cysticercosis is caused by the infection with the larval stage of *T. solium*. The case reported here had thousands of calcifications, which may be caused by the ingestion of many eggs of *T. solium* once or accumulative effects of repeated ingestions. By checking the hospital records, we found another 13 patients with cysticercosis in the town where this patient lived, between 2014 and 2018, but all of those 13 cases belong to NCC, only the patient in this study was extensive disseminated cysticercosis. Disseminated cysticercosis is more common in India. Some studies have suggested that genetic abnormalities may be responsible for a high incidence of disseminated cysticercosis in the Indian population, and they noted that Toll-like receptor 4 gene abnormalities confer genetic susceptibility to disseminated cysticercosis as well [12]. However, due to the limitations of the conditions, we did not detect genetic polymorphism in this patient.

Clinical manifestations of cysticercosis depend upon the location of the cyst, cyst burden and host reaction [10]. Cysts in muscles may manifest as muscular pain, weakness, or pseudohypertrophy; cysts in subcutaneous is frequently asymptomatic but may manifest palpable nodules; and cysts in central nervous system usually cause epilepsy [1, 2, 13, 15]. However, this case did not have the typical symptoms of cysticercosis, only with mild headaches and dizziness, which may be due to the difference in host reactions.

Early detection and diagnosis are important for cysticercosis and taeniasis [20–22]. Early diagnosis and treatment can improve the prognosis of cysticercosis [21, 22]. Early detection and treatment of patients with taeniasis can not only reduce the burden of the patients themselves, but also reduce the risk of the patients as a source of infection to others [20]. Therefore, the application and promotion of advanced diagnostic technology and equipment in endemic area has become particularly important. However, cysticercosis and taeniasis are more prevalent in economically underdeveloped areas, so advanced diagnostic equipment and methods are usually unavailable in some endemic areas [1].

*T. solium* infection is one of the few diseases, which are considered to will be eliminated and eventual eradicated by the International Task Force for Disease Eradication [23]. The following factors make the eradication possible: human beings are the only definitive host; human feces can be effectively controlled; through animal inspection and quarantine, pork containing cysticercosis can be eliminated from the market; infected pigs and humans can be diagnosed through the diagnostic tests for taeniasis and cysticercosis; currently there are effective treatment regimens for taeniasis and cysticercosis [15, 21–23]. Epidemiologic studies have shown that people who live close with cysticercosis have a three times higher risk of serologically positive for cysticercosis than the control group [21]. Thus, it is recommended to screen the case’s family members and the residents in the community, e.g. fecal examinations and serological examinations. If eggs or parasites are detected in the feces, further deworming treatment should be carried out. If the cysticercus antibody is positive, further imaging examination is required to confirm the diagnosis and treatment. Scavenging of food and coprophagy were associated with *T. solium* cysticercosis risk [24] and thus pork should be inspected, and hygiene sanitation should be established including the proper disposal of human feces. Additionally, health education is recognized as an important tool in the control of cysticercosis [25–27], and some studies have shown that health education can effectively reduce human cysticercosis and taeniasis [28, 29]. Thus, health education should be implemented to increase the awareness and then change the raw-eating habits associated with taeniasis and cysticercosis. Regular physical examinations of residents in endemic areas will be beneficial to the early detection of cysticercosis, changes in life style can reduce the risk of taeniasis and cysticercosis, and the attentions and subsequent interventions from health and other departments will facilitate the control and elimination of taeniasis and cysticercosis.

## Conclusion

The life cycle of *T. solium* in the area where the case came from is complete. We expect this case could raise the attentions to the control of *T. solium* infection and subsequent cysticercosis there.

## Data Availability

The data supporting the conclusions of this article are include within the article. Mr. Xin-Zhong Zang is the contact person for Availability of data and materials.

## Abbreviations

CT: Computed tomography
